# Multimodal computed tomography-guided intravenous rtPA for aborted stroke in a HIV-infected young man: a case report

**DOI:** 10.1186/s12879-018-3357-5

**Published:** 2018-08-29

**Authors:** Ping Liu, Min Wu, Ning Zhang, Chunyou Chen, Bing Xiong, Xiaoying Zhang

**Affiliations:** 1 Department of Neurology, First Affiliated Hospital, School of Medicine, Zhejiang University, 79 Qingchun Road, Hangzhou, 310003 China; 2 Department of Neurology, Pujiang People’s Hospital, Pujiang, Jinhua 322200 China; 3 Department of Neurology, the First People’s Hospital of Wenling, Wenling, Taizhou 317500 China; 4 Department of Radiology, First Affiliated Hospital, School of Medicine, Zhejiang University, 79 Qingchun Road, Hangzhou, 310003 China

**Keywords:** HIV infection, Multimodal computed tomography, Stroke, Intravenous thrombolysis

## Abstract

**Background:**

Human immunodeficiency virus (HIV) infection has been recognized as a risk factor for both ischemic and hemorrhagic stroke among young adults. However, information on the optimal management of HIV patients presenting with presumed acute ischemic stroke within the time window of intravenous recombinant tissue plasminogen activator (IV-rtPA) thrombolysis is limited. To the best of our knowledge, the use of multimodal computed tomography (CT)-based imaging to guide acute-phase treatment for patients with HIV infection has never been reported.

**Case presentation:**

We report the clinical, imaging, and immunological features of a young man suffering from presumed acute ischemic stroke, initially without awareness of the presence of HIV infection. IV-rtPA guided by multimodal CT, including brain CT angiography (CTA) and CT perfusion (CTP), was administered at the emergency department. His symptoms were relieved, and there was no recurrence during the 2-month follow up.

**Conclusions:**

Mutimodal CT is a valuable and promising tool for the early management of HIV-infected patients, especially for those presenting within the strict thrombolysis time window.

## Background

Acute ischemic stroke in young adults (under 50 years of age) has been increasingly recognized as a serious health condition. The etiology of stroke in young adults is heterogeneous and differs from that in older patients [1]. Although the pathogenesis of the two conditions differs, HIV infection can reportedly lead to both ischemic and hemorrhagic stroke [2, 3]. Moreover, the optimal approach to diagnostic evaluation and management remains uncertain, particularly for those patients who present within the time window for intravenous recombinant tissue plasminogen activator (IV-rtPA). Due to its convenience and its ability to assess cerebral blood vessels and perfusion at the same time, multimodal computed tomography (CT) is gradually becoming known as a valuable assessment tool for acute stroke patients, as it can provide important information for guiding treatment in the acute phase [4, 5]. In this article, we present a case of IV-rtPA treatment, guided by multimodal CT-based imaging, of an HIV-infected young patient suffering from aborted stroke.

## Case presentation

A 19-year-old male presented to the emergency department at 10:42 a.m.,42 min after the sudden onset of slurred speech with weakness of his right upper and lower extremities. He reported no headache, dizziness, nausea, vomiting, fever, or convulsions. He denied any significant medical history, drug abuse, or high-risk sexual behaviors. He had no history of migraines, trauma, insect bites, exposure to chemicals, or use of medications. Apart from cigarette smoking for 1 year, he denied other risk factors for stroke. There was no history of early cardiovascular disease in the family.

On physical examination, the patient’s vital signs were normal. His weight was 65 kg (69 kg, 3 weeks ago), body mass index (BMI) 21.47 kg/m2. His chest examination was clear, and no additional murmurs were detected upon cardiac examinations. The liver, spleen and cervical lymph nodes were not enlarged; no skin or mucosal lesions were seen.He was alert and oriented to person, place, and time. The pupils were equal and reactive to light and accommodation. He had mild right hemiplegia with strength of 4:5 in the right upper and lower extremities; slight dysarthria and right lower facial paresis were also noted. The neurologic examination was otherwise unremarkable. The National Institutes of Health Stroke Scale (NIHSS) score was 3.

**Fig. 1:**
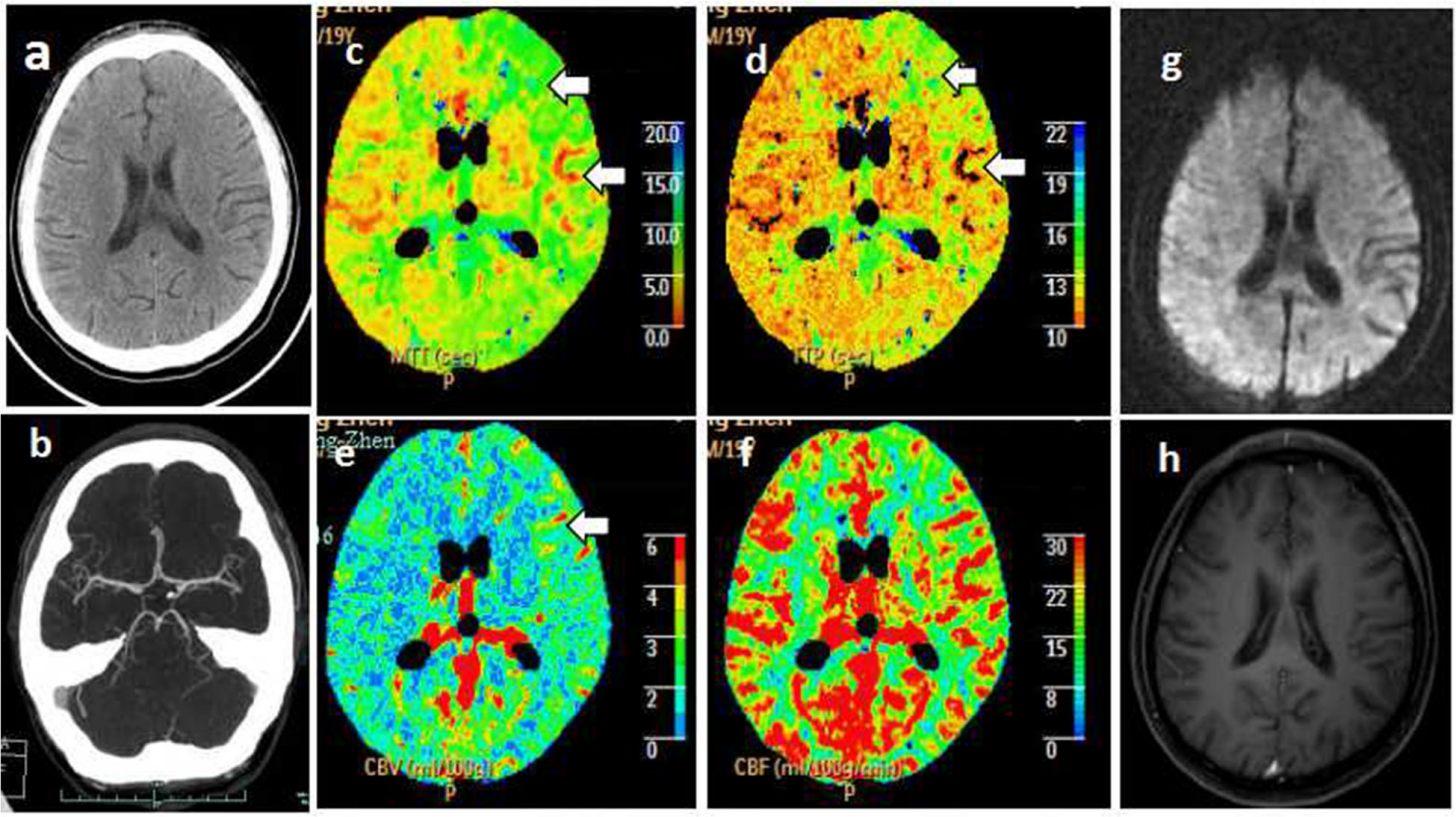
The patient’s neurologic images. **a**–**f** Mutimodal CT obtained 2 h after onset: (**a**) Normal nonenhanced CT. **b** Normal intracranial CTA. **c**–**f** Color maps of CTP showed prolonged MTT (**c**) and TTP (**d**) in the left hemisphere. The corresponding CBV (**e**) was slightly increased, and the CBF (**f**) was relatively preserved. **g** DWI of brain MRI obtained the next day was normal. **h** Brain contrast-enhanced MRI showed no meninges or brain parenchymal enhancement

Rapid blood glucose was in the normal range (6.3 mmol/L). Complete blood count results showed white blood cell (WBC) count 3.0 × 10^9^/L, hemoglobin 11.9 g/dL, and platelets 273 × 10^12^/L. Stroke was first considered. As he was then in the 4.5-h time window for IV-rtPA, an urgent brain CT with computed tomography angiography (CTA) of intra–extracranial vessels and whole-brain computed tomography perfusion (CTP) imaging were performed in the emergency department. The nonenhanced CT (NECT) scan (Fig. 1a) and CTA (Fig. 1b) were normal. CTP showed hypoperfusion in the left hemisphere with prolonged mean transit time (MTT) and time-to-peak (TTP), slightly increased cerebral blood volume (CBV), and relatively preserved cerebral blood flow (CBF) (Fig. 1c-f). A diagnosis of acute ischemic stroke was made. After excluding absolute contraindications for intravenous thrombolysis, 58.5 mg (0.9 mg/kg) rtPA was given at 12:30 a.m., immediately after the patient signed the informed consent. After 1 h of rtPA, his symptoms were alleviated, and the NIHSS score was decreased to 1, with slight asymmetry of the nasolabial sulcus. The patient was then admitted to the department of neurology for further investigations and treatment. Within 60 min after admission, the clinical neurological examination had completely normalized.

On the next day, the patient had no complaints of discomfort. The NIHSS score was 0. Cerebral magnetic resonance imaging (MRI) was performed, and no acute lesion was seen in the diffusion-weighted image (DWI) sequences (Fig. 1g). Doppler ultrasonography of carotidal and intracranial arteries showed no abnormality. Electrocardiogram, electroencephalogram (EEG) and chest CT scan were all normal. Echocardiography showed that the left and right heart chambers were within normal size and function(left ventricle ejection fraction 70%). His laboratory tests showed WBC count 2.2 × 10^9^/L, hemoglobin 10.4 g/dL, platelets 210 × 10^12^/L, ESR 18 mm/h (normal 0–15.0), CRP 0.66 mg/L (normal 0–8.0), homocystein 10.4 umol/L (normal < 15.0), and mild impairment of liver function (ALT 132 U/L (normal 5–40), AST 106 U/L (normal 8–40), GGT 257 U/L (normal 11–50), ALP 127 U/L (normal 40–150)). Serologies showed the following: rapid plasma reagin, negative; antinuclear antibody and antineutrophil cytoplasmic antibody, negative; complement level including C3, C4 levels, normal; anticardiolipin immunoglobulin G(ACL-IgG), ACL-IgM, and ACL-IgA antibody levels, normal; β2 glycoprotein I immunoglobulin G (β2-GP1- IgG) and β2-GP1- IgM antibody levels, normal; high β2-GP I-IgA antibody level, 67.16 units/mL (normal 0–18). HIV enzyme-linked immunosorbent assay and Western blot confirmed HIV infection with CD4 cell count of 7 cells/uL; the CD4:CD8 T cell ratio was 0.04 (7/173). Hepatitis B virus surface antigen and hepatitis C antibody were unremarkable.

On the third day of admission, the patient received a lumbar puncture with pressure of 140 mmH_2_O. Cerebrospinal fluid (CSF) studies showed normal-range white cell and red cell counts but a high protein level at 1185 mg/L. The CSF glucose and chloride were in the normal range. CSF viral PCRs (including herpes simplex virus, varicella zoster, Epstein Barr, cytomegalovirus and JC virus), cryptococcal antigen, and bacterial and fungal cultures were all negative. CSF syphilis TRUST and TPPA tests were also negative. There was no meninges or brain parenchymal enhancement on his brain contrast-enhanced MRI (Fig. 1h).

The patient was diagnosed with aborted stroke and HIV infection. Oral aspirin 100 mg and atorvastatin calcium 20 mg daily were given. For further treatment, the patient was transferred to the HIV/AIDS ward on the fourth day of admission. On follow up 2 months later, he reported no similar symptoms.

## Discussion and conclusions

To our knowledge, this is the first reported case of IV-rtPA stroke treatment guided by multimodal CT imaging in an untreated HIV-infected young adult. This young patient presented at the emergency department with stroke-like manifestations within the time window of intravenous thrombolysis. It has been reported that more than 20% of adults and children with acute stroke-like symptoms will prove to have an alternative diagnosis [1]. Given the potential risks of thrombolytic complications in stroke mimics, especially symptomatic intracranial hemorrhage, rapid and effective assessment is of great importance for disease diagnosis and differentiation as well as for guiding acute management, therapy, and prognosis. Mutimodal CT imaging, which consists of NECT, CTA of the head and neck, and CTP, is now readily available in the emergency department and serves as an ideal tool for rapid image evaluation of stroke and stroke-mimic patients [5, 6]. NECT can effectively exclude hemorrhage as a contraindication for thrombolysis. CTA can define any arterial occlusion or stenosis and collateral blood flow. CTP can assess regional cerebral blood perfusion to adequately delineate the infarct core and the ischemic penumbra (mismatch) [5]. Especially, MTT values can be prolonged not only in stroke but also in transient ischemia attack (TIA), reflecting the high sensitivity for detecting ischemia [7]. For this patient, multimodal CT imaging showed normal NECT and CTA but hypoperfusion in the left hemisphere, which could explain the neurologic deficits and signs at that time. Acute ischemic stroke was first considered. Hence, after excluding the absolute contraindications for thrombolysis, IV-rtPA was given based on certainty that we were working within the time window. After the intravenous thrombolysis, the patient got complete resolution of symptoms with negative DWI imaging. We diagnosed the patient with aborted stroke, since the natural course of the “TIAs” would likely have been strokes [8], especially in setting of IV-tPA use. The other possible causes of transient neurological deficits, such as migraine and Todd paralysis, seemed unlikely given his negative headache history and normal EEG result.

Unexpectedly, the next day’s laboratory tests revealed the presence of HIV infection. Several retrospective studies have reported a positive association between HIV infection and stroke, particularly in young adults without conventional cardiovascular risk factors. Possible HIV-related causes of acute ischemic stroke include opportunistic infection, HIV-associated vasculitis, accelerated atherosclerosis, cardioembolism, combination antiretroviral therapy, and hypercoagulability [3, 9, 10]. For this patient, we found a markedly increased level of β2-GP I-IgA antibody as well as hypoperfusion in the left hemisphere on brain CTP. The other brain imaging examinations, including CTA of the intra-extracranial vessels and contrast MRI, were normal, and there was no evidence of central nerve system infections at that time. The presence of antiphospholipid (aPL) antibodies is closely related to the occurrence of venous and arterial thromboses, thrombocytopenia, abortions, chorea, and a variety of other clinical disorders in patients with autoimmune disorders such as systemic lupus erythematosus [11]. Antiphospholipid antibodies can also be detected in various infectious diseases, such as tuberculosis, syphilis, or viral infections. Recently published studies have suggested the participation of aPL antibodies in the development of thrombotic complications in HIV patients [12, 13]. IgA anti-β2GPI antibodies directed to domain I*V*/V of β2GPI represent an important subgroup of clinically relevant aPLs. Studies have shown that IgA anti-β2GPI antibodies are independent risk factors for acute myocardial infarction and atherosclerosis in populations without antiphospholipid syndrome [14–16], and the same positive association was found for acute cerebral ischemia [17]. Patients with isolated positive for anti-beta2-glycoprotein I IgA had a higher incidence rate for the development of APS events, mainly arterial thrombosis, compared with controls [18]. Moreover, more than 80% of HIV patients with cerebral perfusion abnormalities (focal or diffuse defects in uptake) detected by ^99m^Tc-HMPAO-SPECT were reported to have anticardiolipin antibodies [19]. Thus, we speculated that the increased anti-beta2-glycoprotein I IgA was closely associated with the patient’s stroke symptoms.

Although ischemic stroke is more frequently reported, new evidence suggests that HIV infection may also be an independent risk factor for primary intracranial hemorrhage [20, 21]. Hemorrhagic transformation after thrombolysis occurred in 6% of HIV-infected patients with presumed acute ischemic stroke [2]. HIV-associated vasculopathy (aneurysm or vasculitis), infective vasculitis, infective meningitis, and HIV-associated thrombocytopenia may increase hemorrhage risk. Because HIV testing is not routine in patients with acute ischemic stroke, effective assessment within a short time frame is helpful for deciding whether to give thrombolysis. For our patient, the negative CTA imaging and the low NIHSS score minimized the possibility of hemorrhage risk. The patient did not suffer hemorrhagic transformation, as confirmed by his later brain MRI examination. However, physicians should also be aware of that increase intracranial hemorrhage risk exists in the condition of HIV infection, thus patients should be selected carefully before thrombolysis.

In conclusion, for the first time, we report a case of IV-rtPA use, guided by multimodal CT images, in a HIV-infected young man suffering from presumed acute ischemic stroke. It is important that HIV infection be considered in young adult stroke patients; multimodal CT is an effective and helpful examination tool for acute management of young stroke patents.

## Abbreviations

ACL-IgG: Anticardiolipin immunoglobulin G
aPL: Antiphospholipid
CBF: Relatively preserved cerebral blood flow
CBV: Cerebral blood volume
CSF: Cerebrospinal fluid
CT: Computed tomography
CTA: Computed tomography angiography
CTP: Computed tomography perfusion
DWI: Diffusion weighted image
EEG: Electroencephalogram
HIV: Human immunodeficiency virus
IV-rtPA: Intravenous recombinant tissue plasminogen activator
MRI: Magnetic resonance imaging
MTT: Mean transit time
NECT: Nonenhanced CT
NIHSS: National Institutes of Health Stroke Scale
TIA: Transient ischemia attack
TTP: Time-to-peak
WBC: White blood cell
β2-GP1- IgG: β2 glycoprotein I immunoglobulin G
